# The application of an isotropic crushable foam model to predict the femoral fracture risk

**DOI:** 10.1371/journal.pone.0288776

**Published:** 2023-07-27

**Authors:** Navid Soltanihafshejani, Federica Peroni, Sara Toniutti, Thom Bitter, Esther Tanck, Florieke Eggermont, Nico Verdonschot, Dennis Janssen

**Affiliations:** 1 Radboud University Medical Center, Radboud Institute for Health Sciences, Orthopaedic Research Laboratory, Nijmegen, The Netherlands; 2 Polytechnic University of Milan, Department of Biomedical Engineering, Milan, Italy; 3 University of Twente, Laboratory for Biomechanical Engineering, Faculty of Engineering Technology, Enschede, The Netherlands; University of Vigo, SPAIN

## Abstract

For biomechanical simulations of orthopaedic interventions, it is imperative to implement a material model that can realistically reproduce the nonlinear behavior of the bone structure. However, a proper material model that adequately combines the trabecular and cortical bone response is not yet widely identified. The current paper aims to investigate the possibility of using an isotropic crushable foam (ICF) model dependent on local bone mineral density (BMD) for simulating the femoral fracture risk. The elastoplastic properties of fifty-nine human femoral trabecular cadaveric bone samples were determined and combined with existing cortical bone properties to characterize two forms of the ICF model, a continuous and discontinuous model. Subsequently, the appropriateness of this combined material model was evaluated by simulating femoral fracture experiments, and a comparison with earlier published results of a softening Von-Mises (sVM) material model was made. The obtained mechanical properties of the trabecular bone specimens were comparable to previous findings. Furthermore, the ultimate failure load predicted by the simulations of femoral fractures was on average 79% and 90% for the continuous and discontinuous forms of the ICF model and 82% of the experimental value for the sVM material model. Also, the fracture locations predicted by ICF models were comparable to the experiments. In conclusion, a nonlinear material model dependent on BMD was characterized for human femoral bone. Our findings indicate that the ICF model could predict the femoral bone strength and reproduce the variable fracture locations in the experiments.

## 1. Introduction

For biomechanical simulations of orthopaedic interventions, it is imperative to implement a realistic mechanical response of the bone [1, 2]. Particularly in cases with excessive loading or weak bone strength, the plastic behavior of bone plays a significant role in the mechanical response [3]. Examples of such cases include bone fractures, collapses resulting from mechanical overload, or press-fit fixation of an implant. Finite element analysis (FEA) has proven to be a powerful tool for assessing these types of permanent deformations [4–9].

To capture the failure mechanics, it is crucial to apply an appropriate material model to predict the nonlinear response of trabecular and cortical bone [10, 11], as both can contribute significantly to bone strength [12, 13]. While the cortical bone response can be simulated quite accurately using elastoplastic material models [13], trabecular bone exhibits a more complex nonlinear behavior due to its cellular structure, which is more challenging to simulate in computational modeling [14].

In situations where the trabecular bone plays a more important role, for instance when analyzing the fixation of tibial implants in total knee arthroplasty (TKA) or intervertebral disc arthroplasty, material models such as the isotropic crushable foam (ICF) model can better represent the mechanical response of bone [15, 16]. In situations where the cortex is mainly responsible for the structural function (e.g., in the case of femoral fractures), elastoplastic material models such as the softening Von-Mises (sVM) criterion are very suitable [17, 18]. However, in most cases, both the trabecular and cortical bone simultaneously influence the mechanical response. Unfortunately, using multiple plasticity models in a single FEA simulation is complicated and may cause an undesired interference of the material models, such as inconsistent material behavior at material interfaces, complications in accurately capturing the deformation behavior of the structure, and increased computational costs. This interference may be prevented by combining the mechanical properties of trabecular and cortical bone in a single material model that incorporates the pressure dependency and deviatoric stress in the yield criterion [11, 15, 19].

Our previous study showed that the pressure-dependent ICF model can accurately capture the pre- and post-failure response of trabecular bone [11]. Kinzel et al. [19] demonstrated the possibility of using a volumetric crushable foam model to predict the strength of whole bones (femur and vertebrae). As their FEA model did not include a softening or hardening rule, it was unable to capture the post-yield behavior of the bone. However, the post-yield behavior is particularly of importance when analyzing implant fixation and has a significant effect on the mechanics of the bone-implant interface [14]. An ICF model that combines cortical and trabecular bone and includes hardening-softening rules may therefore present a solution for various orthopaedic applications.

The current paper aims to investigate the possibility of using a crushable foam model dependent on bone mineral density (BMD) for simulating both trabecular and cortical bone. For this purpose, the elastoplastic properties of femoral trabecular bone will be determined through experimental examination of eight cadaveric bones, after which these will be combined with existing cortical bone properties reported in the literature [20]. Subsequently, the appropriateness of this combined material model will be evaluated by simulating femoral fracture experiments that were performed previously [18, 21] and by making a comparison with the results of simulations incorporating the sVM material model.

## 2. Materials and methods

### 2.1. Experimental testing

Experimental testing consisted of mechanical experiments to determine the BMD-dependent parameters for the ICF material model. In addition, proximal femoral fracture experiments were simulated to evaluate the material model. A brief description of these experiments is given here.

#### 2.1.1. Obtaining mechanical properties of the femoral trabecular bone

**Fig 1:**
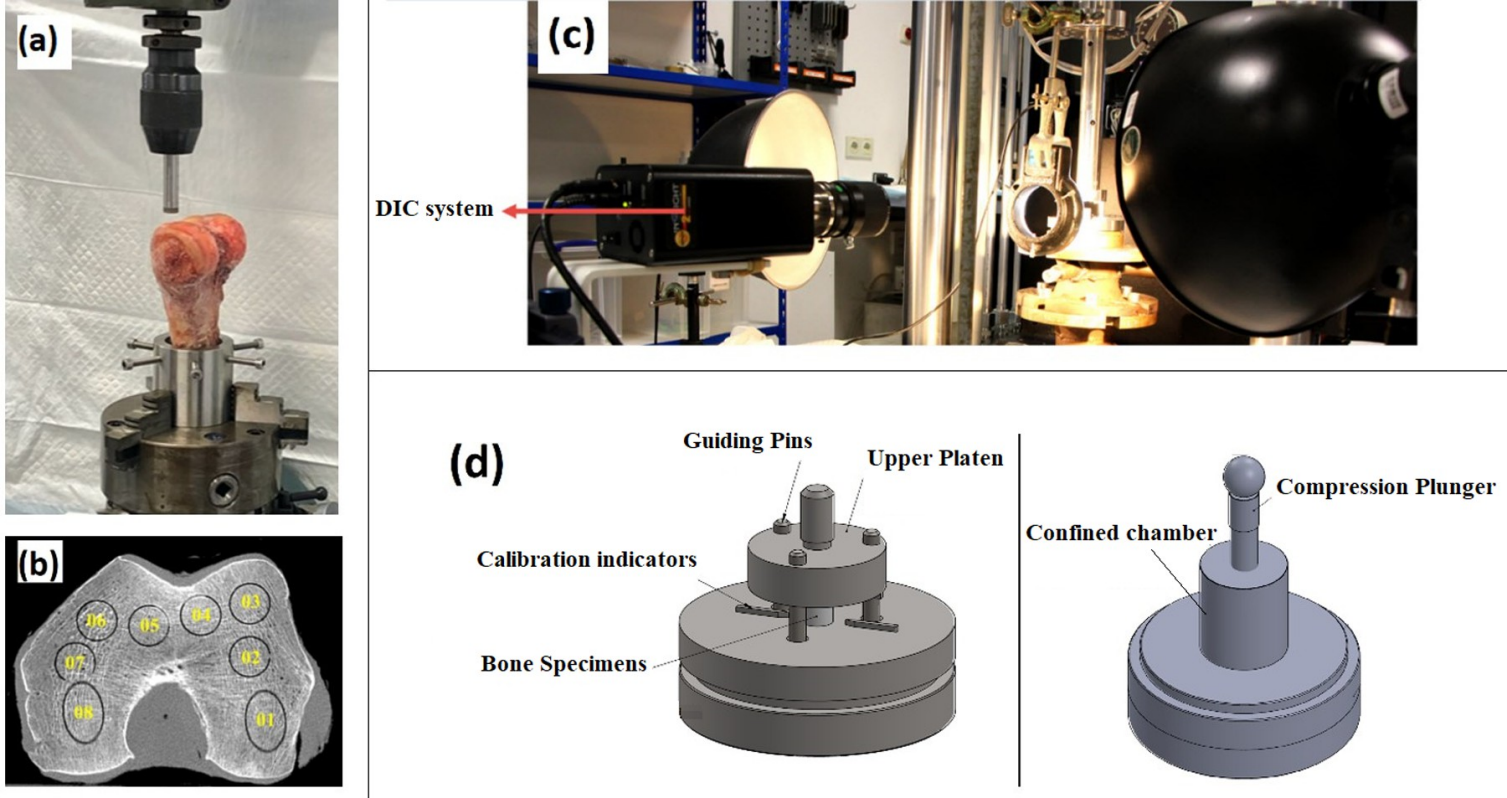
The mechanical experiment of the femoral trabecular bone, (a) Harvesting the specimens (b) Positioning of 8 samples in each bone (c) Experimental setup including DIC system (d) Loading configurations, left is the uniaxial and right is confined setup.

Sixty-four cylindrical samples with a height of 12 mm and a diameter of 11.65 mm were harvested from eight fresh-frozen cadaveric femurs (all male, mean age 72 years, range 60–90 years). Cadaveric Bone are obtained based on Dutch regulation from the Anatomy Department of the Radboud university medical center according to the Dutch Body Donation Program for Science and Education (Wet op de lijkbezorging, hoofdstuk V, artikel 67; 1991) [22]. Body donation of humans aged 60 years and older with a valid hand-written testament was accepted and written consent was obtained from the Head of the Anatomy Department of Radboud university medical center for the use of the cadaveric material in the current study. In order to harvest the cylindrical specimens aligned with the major trabecular orientation, the femora were positioned based on CT scans to adjust the cutting angles in the femoral condyles. Eight samples were taken from the distal part of each femur (**Fig 1A and 1B**). QCT scans of the drilled bones were made prior to removal of the specimens from the distal femur. The scans were captured with a voxel size of 0.4*0.2*0.2 mm (Toshiba Medical Systems, Tokyo, Japan -120 kV-260mA) along with a calibration phantom (0, 50, 100, and 200 mg/ml calcium hydroxyapatite, Image Analysis, Columbia, KY, USA) placed under the bones [23] to obtain Hounsfield Units (HU) of the images. The element-specific HU densities were assigned to the geometrical mesh of each cylinder with a constrained element size of 0.4mm, which were later used in the FEA simulations. As a general representation of the bone density of the full specimen, the HU values of all elements were converted to equivalent BMD values, after which the BMD value was averaged over the elemental values for each cylinder. These average values were used for the definition or the material model.

Following our previously published methods [16], the specimens were divided into two equal groups to perform mechanical testing under uniaxial and confined compression conditions. Bone samples were placed between two parallel custom-made steel platens in uniaxial testing. In confined compression, the test was performed using a plunger and chamber to mimic a hydrostatic state (**Fig 1D**). The compressive load was applied through a ball-joint connection. All samples were subjected to a displacement control force, with a low strain rate of 0.007 s^-1^, approaching quasi-static loading, up to a strain of 0.58 to ensure substantial post-yield deformation.

The bone strains in axial and transverse directions were measured using digital image correlation (DIC) to calculate the Poisson’s ratio in uniaxial compression (**Fig 1C**). As the transverse strains in the confined configuration were equal to zero, only the axial strains were captured to convert the confined principal stresses to a hydrostatic state based on Hooke’s Law. It was assumed that the cylindrical sample deformed axisymmetric before the yielding point (for more details, refer to [11]).

The nominal stress-strain curves of each specimen were created based on the force-displacement data. The linear elastic part of each diagram was identified to calculate the compressive modulus, the Poisson’s ratio in the uniaxial compression, and the yield stress in both the uniaxial and confined conditions. The yield point was based on a 0.2% strain offset. Using Hooke’s law, the confined compression yield stress was converted to hydrostatic stress. Regression analysis was then performed to correlate the mechanical parameters with the average specimen-specific BMD as a power-law equation. The correlations were evaluated using the Pearson’s determination coefficient (*r*^2^).

#### 2.1.2. Proximal femoral fracture

Five fresh-frozen human cadaveric femora, aged from 63 to 96 years old (4 male and one female), were examined previously [18, 21] to assess the failure load and fracture patterns of the proximal femur. Prior to mechanical testing, QCT scan of all femora were captured (ACQSim, Philips, Eindhoven, The Netherlands-120 kVp, 220mAs) with a slice thickness of 3.0 mm and an in-plane resolution of 0.9375 mm. A calibration phantom of hydroxyapatite (Image Analysis, Columbia, Kentucky) was placed under the bones while the images were taken.

**Fig 2:**
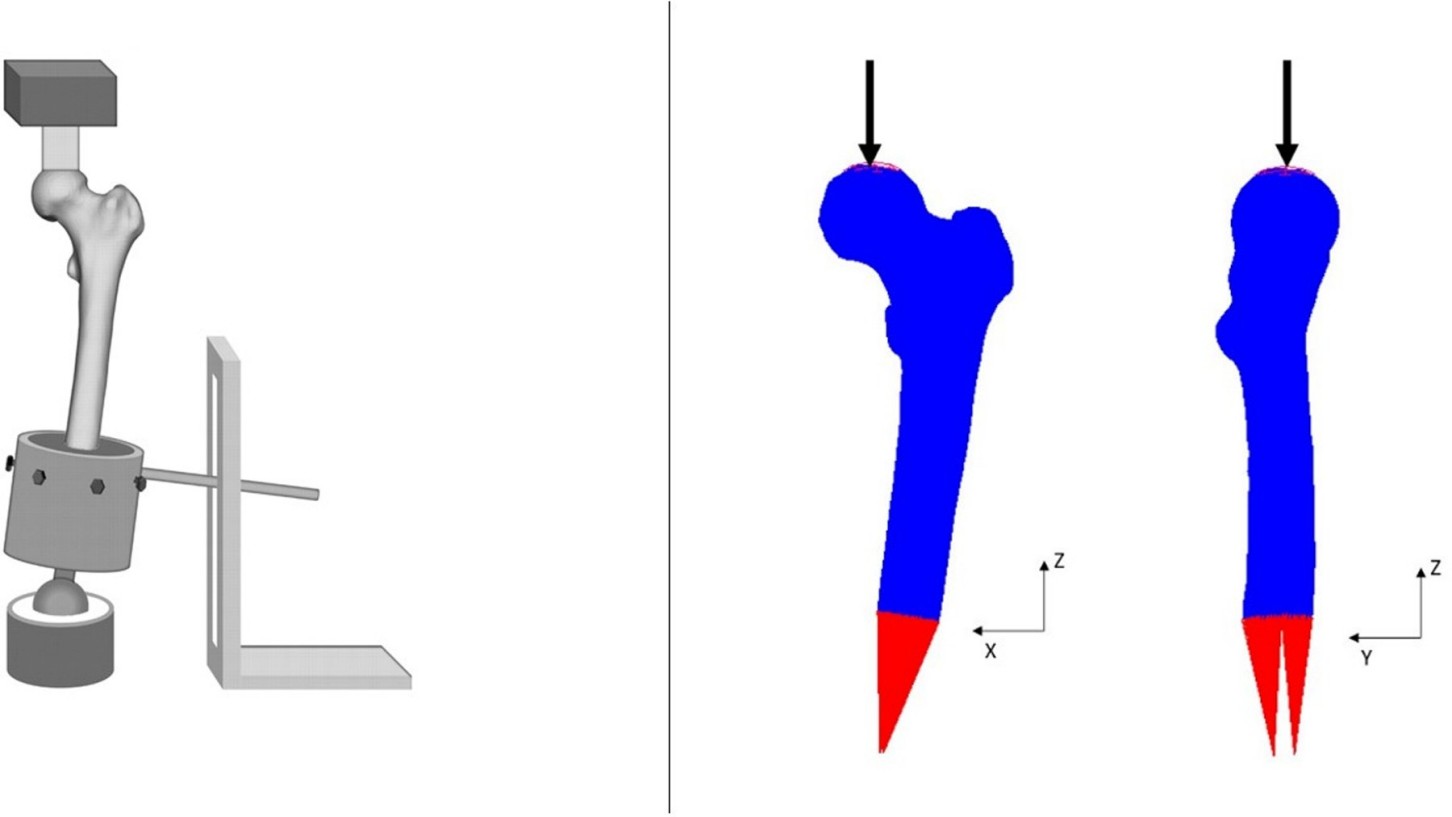
The configuration of the femora in experimental set-up [21] (left ) and FEA simulation(right).

During testing, the femora were placed in a custom-made setup (Fig 2) to restrict all the movements except rotation around the anteroposterior axis [21]. Additionally, to virtually position the femora in the FEA model, 3D coordinate information of the femora were obtained using roentgen stereophotogrammetry analysis (RSA) before loading. The goal of the experiments was to obtain an objective reproducible measurement of the strength of the femur. The orientation of single leg stance was chosen as it was easy to reproduce without a large influence of the anatomical variation between the femurs, and it represents a major loading direction of the femur. In this orientation, an axial force with a rate of 10 N/s was applied on the femoral head until failure.

### 2.2 Material model definition

For the definition of the ICF model, the Young’s modulus, yield stress (both in the uniaxial and the hydrostatic compression), and the elastoplastic Poisson’s ratio need to be determined. The ratio of the uniaxial and the hydrostatic yield stress defines the *K* parameter (strength ratio) of the ICF model. Also, the plastic Poisson’s ratio is dependent on this *K* parameter [24] (**S1 Appendix**).

Similar to our previous study [16], the ICF parameters of the trabecular bone were defined as being dependent on the average specimen-specific BMD. For the solid structure of the cortical bone, on the other hand, it was assumed that the strength ratio remained constant. Therefore, the ICF parameters for cortical bone could be characterized using the values of Young’s modulus and yield stress in the uniaxial compression and a constant value of the *K* parameter. The BMD threshold for cortical bone varied from 400 mg/ml to 1200 mg/ml [25–28]; setting this value to 950 mg/ml satisfied the continuity of the distinctive yield surfaces in the ICF model (**Eq. A1**).

Consequently, as it was assumed that the strength ratio of the cortical structure remained constant, the material properties of the femoral cortical bone reported in previous studies [20, 25, 27] could be used and combined with the trabecular data of the current study. Kaneko et al. [20] provided sufficient details for the experimental samples, and their reported data has been widely used in simulations of femoral fracture [17, 18, 23, 29]. The reported mechanical properties of the cortical bone by Kaneko et al. were obtained from the femoral diaphysis of two male donors.

### 2.3. Numerical simulations

#### 2.3.1. FEA of materials testing with femoral trabecular bone

FEA models were made of the cylindrical femoral trabecular bone specimens to simulate the uniaxial and confined compression experiments for comparison against the experimental results. The FEA models were assigned with the ICF model material properties based on the local element-specific HU values, using the femoral bone parameters. For comparative purposes, simulations were also performed using the sVM criterion. The FEA models were taken from our previous study [16]; for completeness, we here present the most important model features. The FEA models were created based on CT-scans of the trabecular bone specimens. To evaluate the precision of the results, a convergence analysis was performed on a sample with single BMD value using four mesh sizes (0.4, 1, 1.5, and 2 mm). The total strain energy was adopted as the criterion, and convergence was achieved for the first three mesh sizes with differences of less than 10% [11]. After evaluating the mesh convergence results and determining the ideal mesh size for assigning BMD values, a cylindrical model was selected with an element size of 1 mm. The uniaxial loading conditions were simulated through rigid plates at the top and bottom of the specimen, with frictionless contact conditions between the bone and the plates. The experimental displacements were applied to the upper plate, while the bottom plate was fixed in all directions. In the confined condition, in addition, radial expansion was restricted at the outer nodes of the cylindrical specimens to simulate the interaction between the bone and compression chamber. All simulations were performed in MSC.MARC2021 (MSC Software Corporation, Santa Ana, California).

#### 2.3.2. FEA of proximal femoral fracture experiments

The FEA models of five proximal femurs were taken from our previous study [21]; the most important features are given here. FEA models of the femurs were created based on QCT scans that were used to extract the geometry and bone density. The models were created using four-noded tetrahedral elements with an element size of 2 mm in correspondence with our previous work [21]. To replicate the experimental boundary conditions, each femur model was aligned with the experimental setup based on RSA measurements. The distal part of the femur was represented by two sets of high-stiffness springs that were fixed distally. The femoral head was subjected to a displacement-controlled compressive load of 5 mm at 0.1 mm displacement increments. The compressive load was applied using a rigid cup that mimicking the experimental load applicator, which was in frictionless contact with the proximal femoral head. To prevent discretization errors leading to excessive plastic deformations, the layer of bone in direct contact with the load applicator was assigned with elastic bone properties. MSC.MARC2021 (MSC Software Corporation, Santa Ana, California) was used to perform the simulations. A FORTRAN subprogram that integrated user-subroutines of the FEA software was utilized to apply heterogeneous material behavior on the model [30]. Two different constitutive material models were implemented for the assessment of the yielding (and post-yielding) behavior: the sVM and the ICF model. The ultimate failure load and yielding pattern of the proximal femurs were compared in the FEA models against experimental results. A more detailed description of the proximal femur experiments, FEA simulations, and FORTRAN subroutine can be found in previous literature [11, 18, 21].

## 3. Results

### 3.1. Experimental results

**Fig 3:**
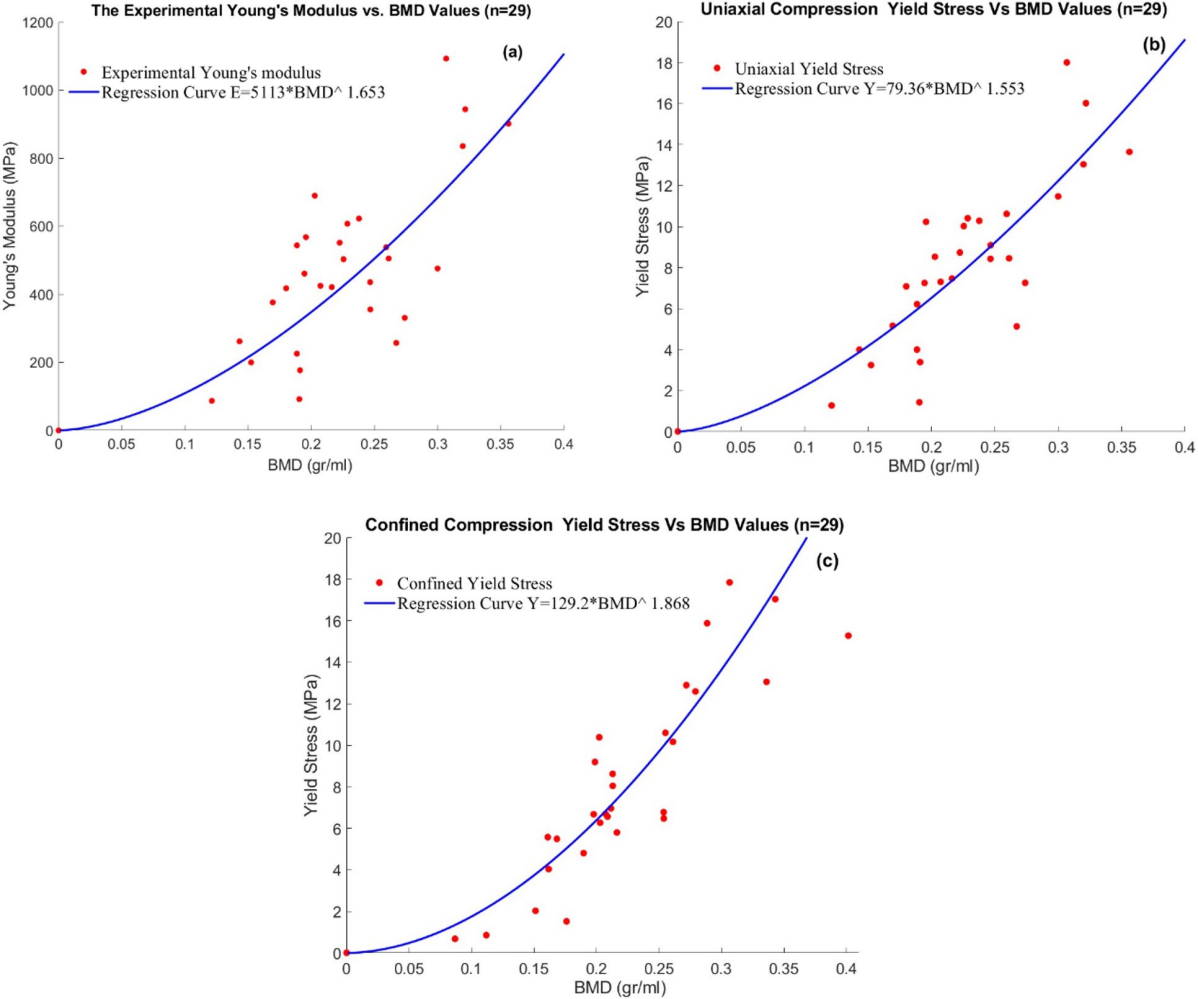
The regression analyses of the measured experimental data for femoral trabecular bone: (a) Youngs’s modulus; (b) Yield stress in the uniaxial compression; (c) Yield stress in the confined compression.

Force-displacement data were collected for 58 out of the 64 cylindrical bone specimens (29 uniaxial and 29 confined tests). No data were obtained for six specimens due to structural failure during the preparation phase. The data was converted to normal stress-strain curves, and the mechanical properties were calculated dependent on BMD. Statistical Analyses showed significant nonlinear correlations for Young’s modulus (*E* ) (*r = 0*.*768*, *p <* .*001* and *SEE = 46*.*76*) (Fig 3A**)** yield stress in uniaxial compression (*r = 0*.*836*, *p <* .*001* and *SEE = 0*.*742*) (Fig 3B) and yield stress in confined compression (*r = 0*.*894*, *p <* .*001* and *SEE = 0*.*873*) (Fig 3C) with the BMD values. The power-law correlations of each parameter are shown in Fig 3.

**Fig 4:**
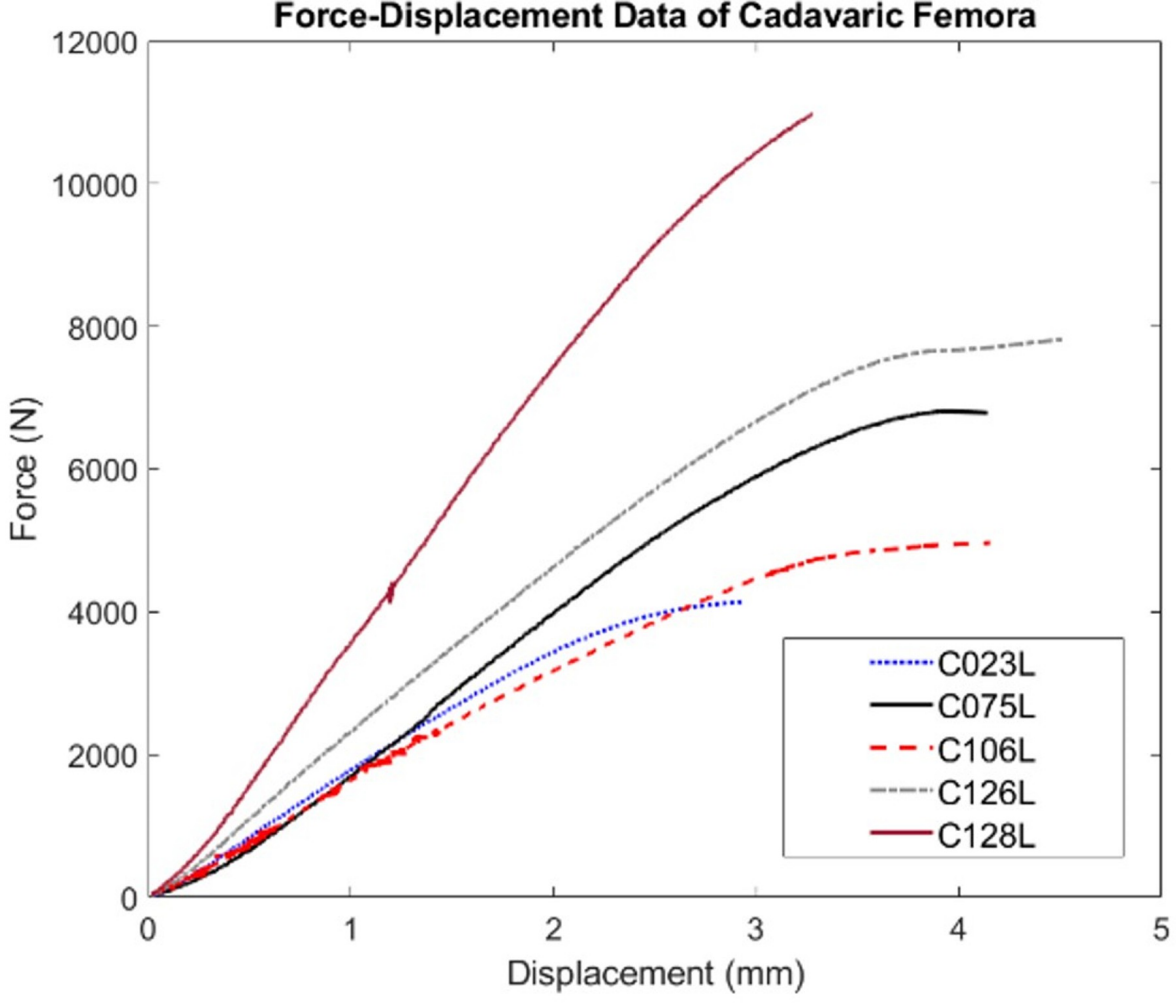
Force-displacement data of five cadaveric femora [18].

The results of the femoral fracture experiments indicate the bone stiffness, maximum compressive force, and displacement at failure **(****Fig** 4 [18]). The maximum failure force ranged from 4,137 N to 10,970 N.

### 3.2. Crushable foam model parameters

As mentioned before, the *K* parameter of the ICF model was defined based on the yield stress in uniaxial and hydrostatic compression, dependent on the BMD. The power-law equation for the *K* parameter was defined as follows:

$$K=a*BMD^{b}$$

with *a = 1*.*361* and *b = -0*.*312*. When the BMD value was above 0.400 gr/ml, the yield surface with a BMD-dependent *K* parameter showed minimal variation from the yield surface with a constant K parameter. This suggests that assuming an unchanged strength ratio (*K* parameter) for cortical bone has no significant impact on the yield surface since the BMD threshold for solid structure of cortical bone is set at a high value of 0.950 gr/ml. According to the physical definition of a cellular structure, the *K* parameter must adhere to 0<*K<3*. Applying the upper border’s limit and considering the constant value for cortical bone, Eq 1 shows the whole range of the *K* parameter for the ICF model:

$$K=\left\{\begin{array}{cc} 2.993 & ,BMD<0.08\\ 1.361\times BMD^{-0.312} & ,0.08<BMD<0.950\\ 1.383 & ,BMD>0.950 \end{array}\right.$$ (1)

In Eq1, K is a dimensionless constant, and BMD represents the bone mineral density in gr/ml.

**Fig 5:**
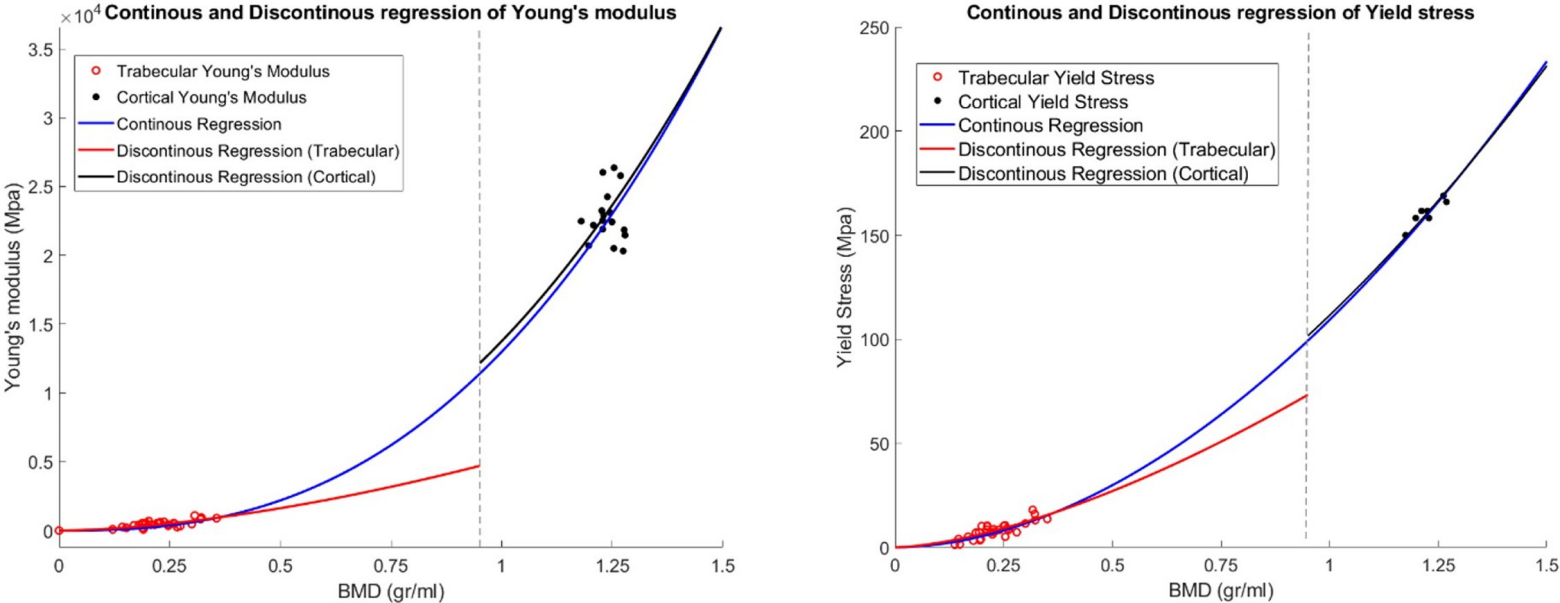
Continuous and Discontinuous regression of Young’s modulus (left) and Yield stress (right). Cortical data was adapted from [20].

Considering the differences in BMD between trabecular and cortical bone, various regression fits were possible on the data, either in a continuous or discontinuous manner. **Fig** 5 illustrates the two fits that were further explored in the simulations of the femoral fracture experiments. In this figure, the trabecular bone properties were obtained in the current study and the cortical data was adapted from Kaneko et al. [20].

The empirical power relation of the material parameters based on the BMD value concerning **Fig** 5 is as follows in **Table 1**:

**Table 1:** Power-Law equations of the material parameters in continuous and discontinuous approaches (BMD [gr/ml]).

Mechanical Parameters	Continuous	Discontinuous
Compressive stiffness (E) (MPa)	$12980\times \rho_{BMD}^{2.567}$ (*r =* .*745*, *p <* .*001* and *SEE = 59*.*24*)	$5113\times \rho_{BMD}^{1.653},\rho_{BMD}\leq 0.950$ (*r =* .*768*, *p <* .*001* and *SEE = 46*.*76*)
		$13750\times \rho_{BMD}^{2.429},\rho_{BMD}>0.950$ (*r =* .*725*, *p <* .*001* and *SEE = 67*.*43*)
Yield Stress in uniaxial condition (MPa)	$109.3\times \rho_{BMD}^{1.872}$ (*r =* .*881*, *p <* .*001* and *SEE = 0*.*450*)	$79.36\times \rho_{BMD}^{1.553},\rho_{BMD}\leq 0.950$ (*r =* .*836*, *p <* .*001* and *SEE = 0*.*742*)
		$111.5\times \rho_{BMD}^{1.800},\rho_{BMD}>0.950$ (*r =* .*887*, *p <* .*001* and *SEE = 0*.*361*)
Elastic Poisson’s ratio	0.16	
Plastic Poisson’s ratio	$\frac{3-K_{\left(BMD\right)}^{2}}{6}$	

r = Spearman’s rho; p = probability; SEE = Standard Error of the Estimation

### 3.3. Numerical simulation

**Fig 6:**
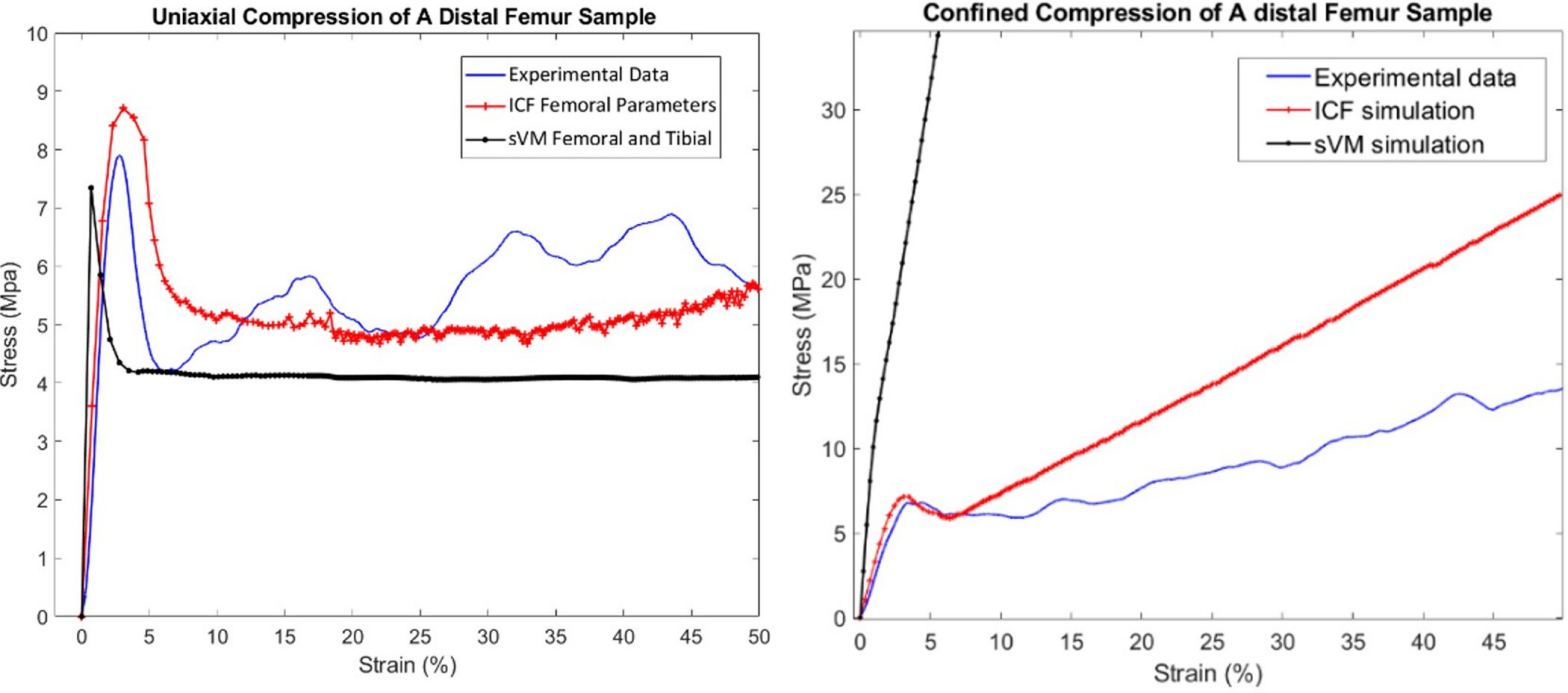
Stress-strain data of a sample with BMD of 207.5 mg/ml in uniaxial simulation (left) and a BMD of 208.3 mg/ml in confined simulation (right) with two different material models versus experimental results.

The ICF model and sVM material models were applied to the FEA simulation of the cylindrical bone specimens in uniaxial and confined compression. In uniaxial compression, the numerical stress-strain curves from the ICF model were very similar to the experimental data. However, in the sVM simulations, the stiffness was overestimated, resulting in an underestimation of the yield strain. In the simulations of confined compression, the stiffness, yield stress, and ultimate stress were accurately simulated with the ICF model. The sVM overpredicted the stiffness and could not reproduce the yield point. **Fig** 6 shows the experimental and computational stress-strain curves for two typical samples tested under uniaxial (BMD of 207.5 mg/ml) and confined conditions (BMD of 208.3 mg/ml). The maximum stress value at the end of the confined simulation (50% of strain) with the sVM criterion was 280 MPa, while this value was 25 MPa for the ICF model and 14 MPa in the experimental data.

**Fig 7:**
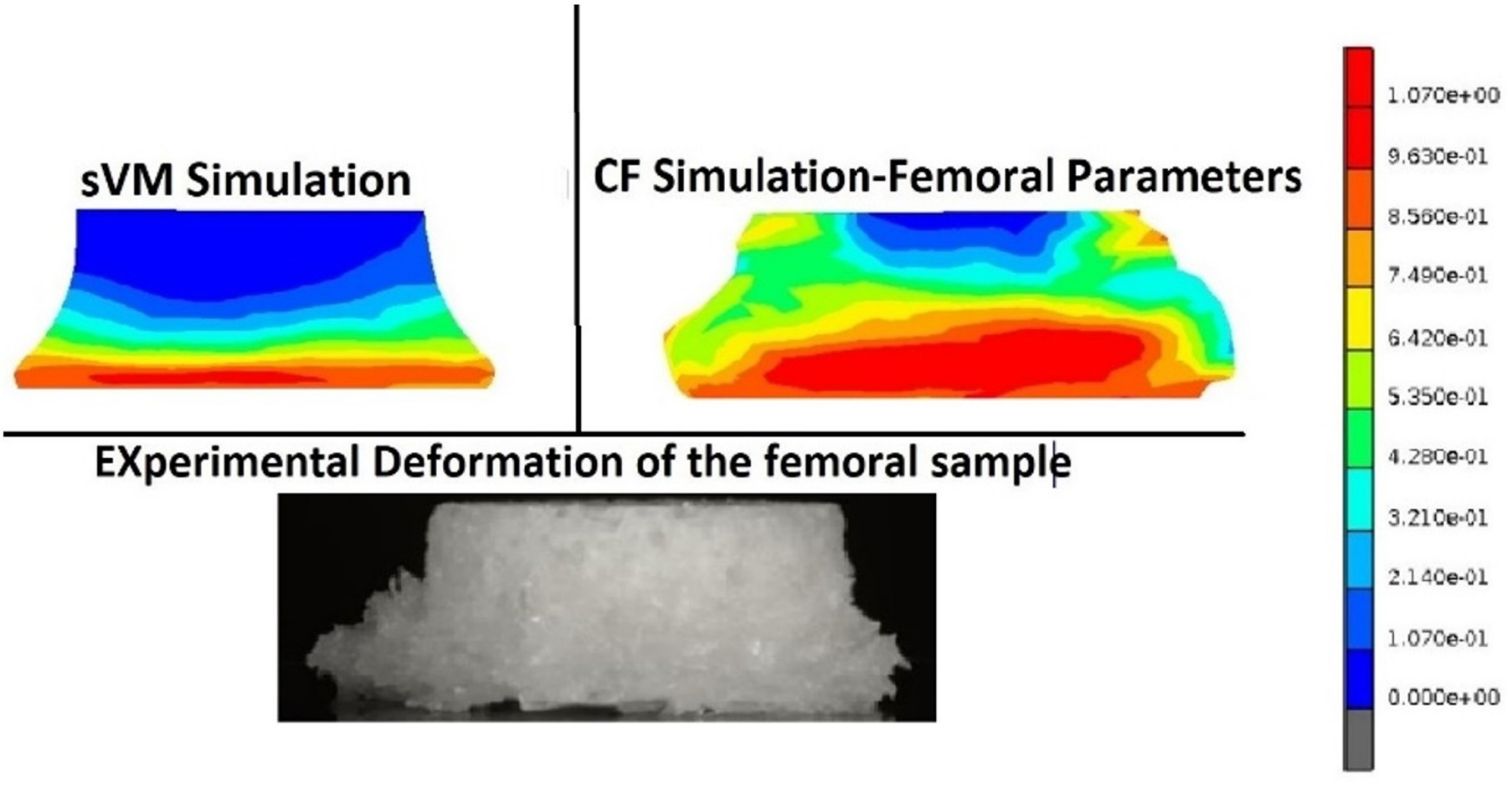
The equivalent plastic strain represents permanent deformation of a femoral sample with a BMD of 207.5 mg/ml.

The equivalent plastic strain (EPS) distribution of a bone sample (BMD 207.5 mg/ml) under uniaxial compression is shown in **Fig** 7. In contrast with the sVM model, the distribution of plastic deformation in the ICF simulation compared relatively well with the deformations observed in the experiments.

**Fig 8:**
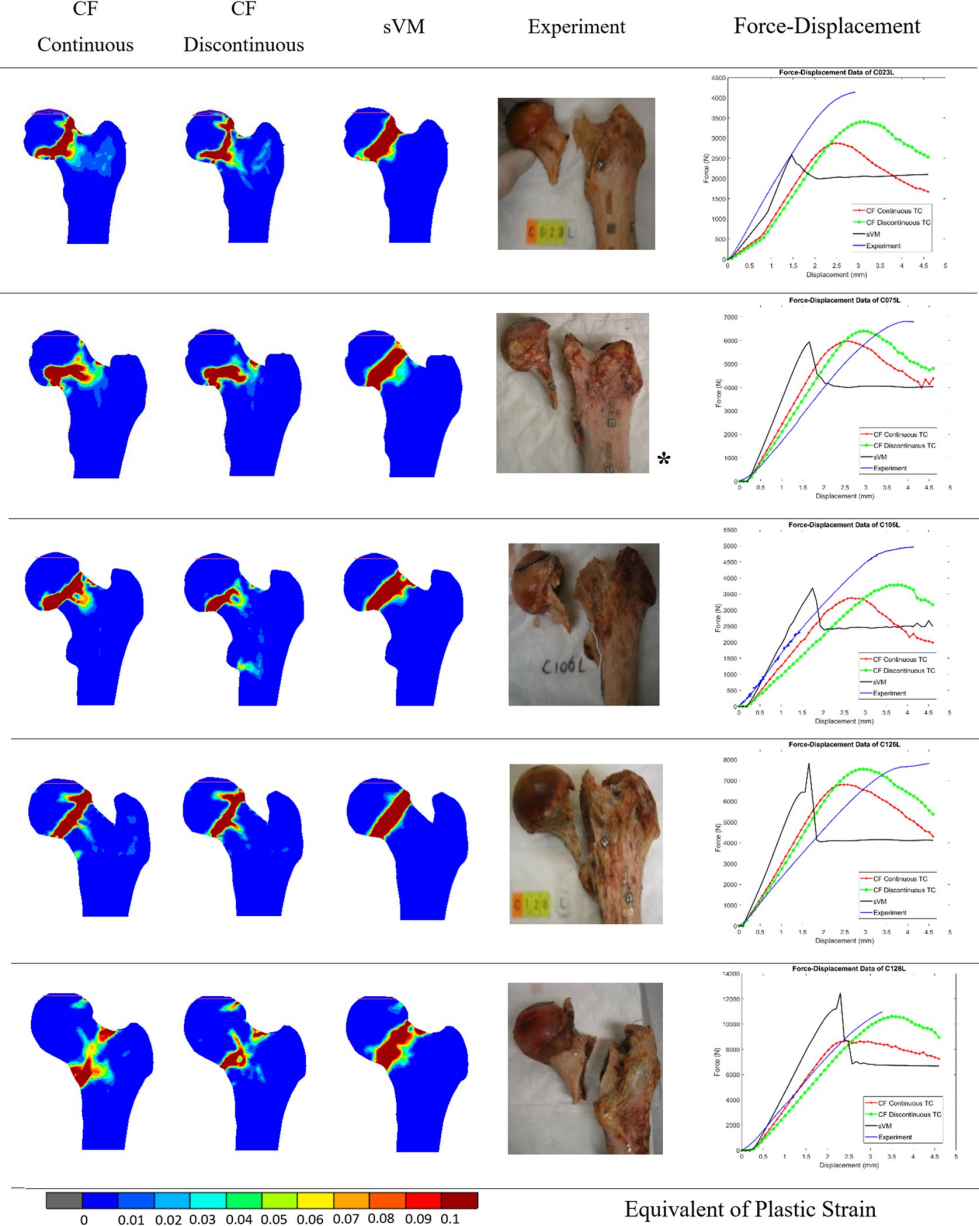
Comparison between the distributions of the equivalent plastic strain, indicating the fracture locations of the FEA models and the actual fracture location in experiment. The graphs on the right show the force-displacement data of the FEA models and physical experiments. *Images of cadaveric specimens were adapted from [18].

The ICF (with continuous and discontinuous fits) and the sVM material models were subsequently applied in FEA simulations of the fracture experiments. Fig 8 lists the distributions of EPS, indicating the fracture locations of the FEA models, and the force-displacement data, representing the mechanical response of the bones in the simulations and experiments. The ICF model with the continuous and discontinuous fits predicted experimental failure load with an average accuracy (percentage of estimated failure load to the experimentally measured failure load) of 79% and 90%, respectively, while the sVM criterion predicted the ultimate failure load with an accuracy of 82%. Although both ICF models demonstrated a comparable stiffness of the proximal femur, the sVM resulted in a stiffer bone behavior. The sVM model provided an average accuracy of 5% for the predicted stiffness compared to the experimental values, with an over-prediction factor of 2. However, the ICF model exhibited a higher average accuracy in predicting stiffness, with 80.5% and 85.5% accuracy for the continuous and discontinuous models, respectively.

## 4. Discussion

In the current study, we investigated the feasibility of using an ICF model that combines human trabecular and cortical bone properties to predict the mechanical response of the femur. For this purpose, data from material tests with trabecular bone were combined with cortical bone properties taken from the literature. For validation purposes, the characterized ICF model was subsequently used to simulate fracture tests of five cadaveric proximal femurs from a previous study.

The mechanical properties of the trabecular bone specimens were comparable to previous findings [16]. The compressive stiffness of current specimens was in the range of 100–1100 MPa, similar to values reported by Rho et al. (150–300 MPa) and Goldstein (7.6–1516 MPa) [31, 32]. The uniaxial yield stress of the current samples varied from 1 MPa to 18 MPa, which was within the range of 1.64–15.3 MPa measured by Kaneko et al. [33]. The compressive strength of 0.16–18 MPa for confined compression was consistent with the values reported by Carter et al. [34] and Charlebois et al. [35] (0.24–31.59 MPa). In uniaxial and confined compression, the Young’s modulus and yield stress were very similar to the values for the tibial trabecular bone that we found previously [16]. However, due to the different BMD ranges, the regression analysis showed different power-law equations, leading to a different value for the *K* parameter. Consequently, simulations with tibial and femoral ICF parameters would lead to different but yet comparable results.

Simulations of the trabecular bone experiments demonstrated that, although both material models provided a reasonable prediction for the uniaxial compression tests, the sVM model resulted in a significant overestimation of the stiffness and yield stress under confined compression, likely due to the different properties used in the sVM criterion [17]. In the sVM model, the material properties of femoral trabecular and cortical bone were based on values reported by Kaneko et al. [20, 33], with the trabecular properties being determined at a higher strain rate compared to the current study. On the other hand, another cause for the overprediction of stiffness and yield strength in the confined configuration may be that the sVM model does not consider hydrostatic stress in the yield criterion as opposed to the ICF model that was able to capture partial softening in the confined simulations.

A truly rigid confinement hardly occurs during physiological loading in human bone [16]. However, the trabecular bone is surrounded by the much stiffer cortical bone, which in some situations can result in a low-level confinement [5, 15]. For instance, during press-fit implantation of TKA the trabecular bone in the distal femur or the proximal tibia are confined by stiff cortical bone and the stiff implants, resulting in large compressive stresses [5]. A constitutive law that neglects the pressure dependency in the yield criterion may not be suitable for such situations [5, 15]. Obviously, additional research is required to investigate whether the current ICF model would provide a better representation of the bone response in such cases.

The ultimate failure load predicted by the simulations of the femoral fractures was on average 79% (3400–8550 N) and 90% (3810–10831 N) for the continuous and discontinuous ICF material models and 82% (3700–12370 N) of the experimental value for the sVM model. The differences between the continuous and discontinuous ICF models were likely due to the differences in material mapping; in the range from 0–350 mg/ml the yield stress was higher in the continuous than in the discontinuous fit, while this was opposite in the 350–950 mg/ml BMD range. The similarity of the failure loads predicted by the ICF and sVM material models indicates that although the level of the failure load is dependent on the yield criterion, the actual yield stress value has a significant impact on the overall strength.

Fracture locations in the experiments varied and included the femoral neck (sub-capital), transcervical, and vertical femoral-neck fracture [37, 38]. The fracture locations predicted by the two ICF models were similar and comparable to the experiments, while several other FEA studies tend to predict sub-capital fractures only [13, 17, 29, 36, 37]. Likely, the pressure-dependent yield criterion and updating of the distinctive yield surface facilitated the prediction of these various fracture locations in ICF models. In contrast to the ICF models, the sVM predicted a subcapital fracture in all cases, which was in line with previous studies in which a yield criterion without pressure dependency was used [17, 29]. This difference may result from the fact that plastic behavior in the constitutive formulation of sVM model is dependent on the constant yield criterion, which is not updated after the softening part of the plastic region. However, the mechanism for the prediction of plastic deformation in different constitutive laws is complex and cannot be identified easily.

Kinzel et al. [19] performed a similar study in which femoral fracture was simulated in a stance loading configuration. They demonstrated that a volumetric CF model could predict bone strength and deformation. Also, they compared the volumetric CF model with a sophisticated elastoplastic damage model developed by Garcia et al. [38]. The fracture area predicted in their FEA model was comparable to the experiment, both for the CF model and the sophisticated constitutive law. However, both models could only predict the failure load up to an accuracy of 59%. Kinzel et al. did not include hardening or softening parameters in their FEA models, and the CF model was applied with a constant *K* parameter independent of bone density. The current study illustrates the importance of these factors when simulating femoral fractures.

Limitations of the current study include minor errors in the experiments such as sample preparation, end effects of the platens, and the assumption of axisymmetric deformation of the sample prior to the yielding, as explained previously [16]. Our modeling approach assumed an isotropic bone response, ignoring anisotropy due to the trabecular bone architecture and collagen fibers in the cortical bone, which may have influenced the fracture predictions. Moreover, while several studies have reported mechanical testing results of the femoral bone, the cortical bone properties used in the current ICF models were based on the study by Kaneko et al. [20, 33]. This dataset was chosen as it is the basis of the sVM model by Keyak et al. [17], which is also used at our lab for assessing the femoral fracture risk in patients suffering from metastatic lesions [17, 18, 21]. Using the same dataset for both models ensured a clean comparison of the material models. However, it is likely that using a different source for the cortical bone properties would have affected the outcome of the femoral fracture simulations. Combining multiple datasets may therefore increase the robustness of the material model.

Similarly, in the femoral fracture experiments, the measured displacements likely included some laxity in the experimental setup, which may have resulted in an overestimation of the deformation of the femur, and as a result, underestimation of the structural stiffness [18]. Great care was taken to reproduce the experimental boundary conditions as closely as possible. The femur alignment in the experimental set-up was therefore reproduced in the FEA models based on 3D RSA measurements of the orientation of the femur relative to the loading set-up. The load applicator was represented by a rigid cup, while the distal fixation was applied to springs that were used to artificially elongate the model while reducing the actual number of elements. The boundary conditions applied distally to the springs represented the rotational degrees of freedom in the experimental set-up. Moreover, tests were performed with cadaveric bone, which may behave differently from *in vivo* bone [39]. To ensure an approximation as close as possible to *in vivo* situation, we only used fresh-frozen cadaveric samples, and minimized the number of thawing-freezing cycles and the time the tissue was exposed to room temperature.

## 5. Conclusion

The current study aimed to develop an ICF model that combines trabecular and cortical bone properties, and to investigate whether it can predict the mechanical response of a whole femur. Simulations of compressive tests with trabecular bone specimens indicated the ICF model could accurately reproduce experimental results, even in the case of confined compression. The ICF model was able to predict the femoral bone strength similar to the previously used sVM criterion. Moreover, the ICF model was able to reproduce various fracture locations and orientations observed in the experiments. As such, the ICF model is a promising tool for the evaluation of the femoral fracture risk, but also potentially for applications such as press-fit fixation of TKA components, peri-prosthetic fractures, and collapse of joint reconstructions.

## Supporting information

S1 Appendix Yield surface definition of the isotropic crushable foam model. (DOCX)
