# Supplementary material for: The application of an isotropic crushable foam model to predict the femoral fracture risk
Source: PLoS One. 2023 Jul 27;18(7):e0288776. doi: 10.1371/journal.pone.0288776 (PMC10374151; doi:10.1371/journal.pone.0288776)
Supplement: S1 Appendix — (DOCX) [file pone.0288776.s001.docx]

**Appendix**

The elliptical yield surface of the CF model with isotropic hardening ($F_{ICF}$), which extends alongside the axes of the $p-q$ plane, is given by:

| $F_{ICF}=\sqrt{q^{2}+a^{2}p^{2}}-B$; | (A.1) | $B=ap_{c}=\sigma_{uc}\sqrt{1+{(\frac{a}{3})}^{2}}$ | (A.2) |
| --- | --- | --- | --- |

where $B$ is the size of the q-axis of the yield ellipse,$\sigma_{uc}$ is the absolute compressive strength under uniaxial loading and $a$ is the shape factor of the yield ellipse and is defined as:

| $a=\frac{3K}{\sqrt{9-K^{2}}}$; | (A.3) | $K=\frac{\sigma_{uc}^{0}}{p_{c}^{0}}$ | (A.4) |
| --- | --- | --- | --- |

In these equations 𝐾 is the compressive yield stress ratio, and $p_{c}^{0}$ and $\sigma_{uc}^{0}$ are the initial yield stress under hydrostatic and uniaxial compression conditions, respectively. The plastic Poisson’s ratio can be defined as follows :

$v_{p=}\frac{3-K^{2}}{6}$; (A.5).
